# Outcome of Stem Cell Transplantation in HTLV-1-Associated North American Adult T-Cell Leukemia/Lymphoma

**DOI:** 10.1007/s44228-023-00032-y

**Published:** 2023-03-15

**Authors:** Abdul-Hamid Bazarbachi, Daniel Reef, Hiba Narvel, Riya Patel, Rama Al Hamed, Sindhu Vikash, Karun Neupane, Eleftheria Atalla, Astha Thakkar, Shafia Rahman, Urvi Shah, Diego Adrianzen-Herrera, Ryann Quinn, Sumaira Zareef, Emma Rabinovich, Alyssa De Castro, Felisha Joseph, Kailyn Gillick, Jennat Mustafa, Fariha Khatun, Amanda Lombardo, Latoya Townsend-Nugent, Michelly Abreu, Nicole Chambers, Richard Elkind, Yang Shi, Yanhua Wang, Olga Derman, Kira Gritsman, Ulrich Steidl, Mendel Goldfinger, Noah Kornblum, Aditi Shastri, Ioannis Mantzaris, Liza Bachier-Rodriguez, Nishi Shah, Dennis Cooper, Amit Verma, Bihui Hilda Ye, Murali Janakiram, Roberto Alejandro Sica

**Affiliations:** 1 Internal Medicine Department, Jacobi Medical Center, Albert Einstein College of Medicine, NYC, New York, NY USA; 2 Oncology Department, Montefiore Medical Center, Albert Einstein College of Medicine, NYC, New York, NY USA; 3 Internal Medicine Department, UCSF, San Francisco, CA USA; 4 Oncology Department, Roswell Park Comprehensive Cancer Center, Buffalo, NY USA; 5 The Blood and Marrow Transplant Group of Georgia, Atlanta, GA USA; 6 Department of Cell Biology, Albert Einstein College of Medicine, NYC, New York, USA; 7 City of Hope Comprehensive Cancer Center, Duarte, CA USA

**Keywords:** Allogeneic stem-cell transplantation, Autologous stem-cell transplantation, Adult T-cell leukemia/Lymphoma, Human T-cell lymphotropic virus type I, Hispanic, Black, Minority, North American

## Abstract

Adult T-cell leukemia/lymphoma (ATLL) remains challenging to treat and has dismal outcome. Allogeneic stem-cell transplantation (allo-SCT) has promising results, but data remain scarce. In this single-center retrospective analysis of 100 patients with ATLL from north America (67 acute, 22 lymphomatous), 17 underwent allo-SCT and 5 autologous SCT (ASCT), with a median follow-up of 65 months. Post-transplant 3-years relapse incidence (RI) and non-relapse mortality (NRM) were 51% and 37%, respectively, and 3-year progression-free survival (PFS) and overall survival (OS) were 31% and 35%, respectively. ASCT 1-year RI was 80% compared to 30% in allo-SCT (*p* = 0.03). After adjusting for immortal-time bias, allo-SCT had significantly improved OS (HR = 0.4, *p* = 0.01). In exploratory multivariate analysis, patients achieving first complete response and Karnofsky score ≥ 90 had significantly better outcomes, as did Black patients, compared to Hispanics, who had worse outcome. In transplanted patients, 14 died within 2 years, 4 of which ASCT recipients. Our data are the largest ATLL transplant cohort presented to date outside of Japan and Europe. We show that allo-SCT, but not ASCT, is a valid option in select ATLL patients, and can induce long term survival, with 40% of patients alive after more than 5 years.

## Introduction

Adult T-cell leukemia/lymphoma (ATLL) is a rare mature T cell malignancy, driven by the human T-cell lymphotropic virus type I (HTLV-1), and associated with a grim prognosis [1–3]. HTLV-1 mediates T cell transformation and clonal expansion, resulting in malignant transformation in around 1–4% of the estimated 20 million infected hosts from endemic regions such as southern Japan, the Caribbean, Central and South America, as well as sub-Saharan Africa, Romania and northern Iran [1–4].

Four different subtypes are described by the widely used Shimoyama classification, including smoldering, chronic, and the aggressive acute and lymphomatous subtypes. These subtypes not only differ in their clinical presentations, but also have different outcomes and require distinct treatment strategies [5]. Smoldering and chronic subtypes are associated with better short-term but poor long-term outcome, whether treated with chemotherapy or a watch and wait approach, [6]. However, aggressive subtypes are associated with dismal short-term prognosis, although lymphomatous ATLL has been shown to respond to combination chemotherapy [7]. The combination of zidovudine and IFN-alpha significantly improved first line response rates in the acute subtype [8], as well as smoldering and chronic ATLL which are, otherwise, unresponsive to chemotherapy, with some benefit of its use in lymphomatous ATLL as well [9].

Furthermore, ATLL (NA-ATLL) has been proposed as a separate entity from Japanese ATLL, with worse outcome and particular chemo refractoriness. While specific strategies, such as epigenetic therapies, might be particularly effective [10, 11], relapse rates remain extremely high, making stem-cell transplantation (SCT) an attractive, potentially curative approach.

The use of autologous-SCT (ASCT) has been reported in limited case series, was consistently found to lead to high relapse rates and poor outcome, and is not currently recommended [12–14]. On the other hand, the use of allogeneic-SCT (allo-SCT) has proved to be challenging in these patients, as they usually have poor performance status and rarely achieve complete response (CR), and often lack suitable donors [15]. When feasible, however, allo-SCT with either myeloablative (MAC) [16–18] or reduced-intensity (RIC) [19] conditioning can lead to favorable long-term outcomes, as suggested by multiple reports. These include a large study with 386 patients from Japan, which demonstrated a 3-year overall survival (OS) of 33% [20], and a long-term report of 30 patients receiving RIC, which demonstrated 5-year OS and progression-free survival (PFS) of 36% and 31%, respectively [21]. The long-term favorable outcome after allo-SCT that is not observed following ASCT is likely attributed to the graft-versus-leukemia (GVL) effect, as long term responders usually have higher incidence of chronic graft-versus-host-disease (GVHD) and donor lymphocyte infusion (DLI) use, which leads to durable remissions [22, 23].

Given the data scarcity, we aimed to further elucidate the role of transplant in ATLL, describing patient outcomes post-transplant versus no transplant, and identifying responders’ characteristics. Our results support existing evidence in the literature on the positive role of transplant in ATLL and present novel findings not previously described.

## Materials and Methods

### Study Design and Data Collection

This is a retrospective single center analysis. Included in this analysis are all patients with ATLL treated at the Montefiore Medical Center between 2003 and 2022, who received either allo-SCT from an HLA-matched sibling related donor (MSD), unrelated (MUD), haploidentical (haplo) or cord blood (CB) donor with bone marrow (BM), peripheral blood (PB) or CB stem cells, ASCT, or no transplant. Patient-specific variables collected included age, gender, ethnicity, cytomegalovirus (CMV) and HTLV1 serologies, Karnofsky performance score (KPS), diagnosis date, disease subtype, number of prior therapy lines, status at transplant, transplant date, and prior SCT. Donor and transplant-related variables included donor gender, CMV and HTLV1 serologies, stem cell source, donor type, in-vivo T-cell depletion, engraftment, conditioning regimen, GVHD prophylaxis, maximum acute and chronic GVHD grade, relapse date when applicable, and main cause of death.

### Definitions

MAC was defined as a regimen containing either TBI with a dose ≥ 8 Gy, a total dose of oral busulfan (Bu) > 8 mg/kg, or a total dose of intravenous Bu > 6.4 mg/kg. All other regimens were defined as RIC [24]. Diagnosis and grading of acute [25] and chronic GVHD [26] were performed using standard criteria.

### Endpoints

Endpoints included PFS, OS, non-relapse mortality (NRM), relapse incidence (RI), acute and chronic GVHD, and GVHD-free, relapse-free survival (GRFS), with outcomes measured either from the time of SCT or diagnosis when comparing transplant to non-transplant patients. PFS was defined as survival without disease relapse or progression, with patients censored at the time of last contact. OS was defined as the time until death from any cause, or discharge to hospice with unknown date of death. NRM was defined as being alive until death with no previous relapse. GRFS was defined as being alive with neither grade III-IV acute GVHD, extensive chronic GVHD, nor relapse [27]. Patients who did not experience an event were censored at the date of last contact.

### Statistical Analysis

The Kaplan–Meier method was used to calculate the probabilities of OS and PFS. Cumulative incidence functions were used to estimate RI and NRM in a competing risk setting. Death and relapse were considered as competing events for acute and chronic GVHD.

Univariate analyses were done using the Gray’s test for cumulative incidence functions and the log rank test for OS, GRFS, and PFS. A Cox proportional hazards model was used for multivariate regression, and results were expressed as hazard ratio (HR) with a 95% confidence interval (CI). All tests were two sided. The type-1 error rate was fixed at 0.05 for determination of factors associated with time-to-event outcomes.

To account for immortal time bias, a landmark analysis was performed whereby 33 non-transplanted patients with early mortality within 4 months of diagnosis who were not able to make it to transplant were excluded from the Kaplan–Meier survival and multivariate analysis. The 4-months cutoff was chosen as being the earliest transplanted patients made it from diagnosis to transplant.

All analyses were performed using R version 3.4.0 (R Core Team. R: a language for statistical computing. 2014. R Foundation for Statistical Computing, Vienna, Austria).

## Results

### Patient Characteristics

One hundred patients (57% female; median age 58.5 years [range 18–87]; 69% Blacks and 27% Hispanic) met inclusion criteria (Table 1). Sixty-seven percent had acute ATLL, 22% lymphomatous disease, and 11% missing.

**Table 1:** Patient characteristics

Patient characteristics	Auto *n* = 5	Allo *n* = 17	No transplant *n* = 78	Total *n* = 100	*p* value
	*N* (%)/Median (Range) [IQR]	*N* (%)/Median (Range) [IQR]	*N* (%)/Median (Range) [IQR]	*N* (%)/Median (Range) [IQR]	
Age at diagnosis	53 (45–73) [50–56]	56 (18–74) [42–65]	60.5 (25–87) [44–69.75]	58.5 (18–87) [43.75–69]	0.51
Gender					
Male	2 (40%)	10 (58.8%)	31 (39.7%)	43 (43%)	0.33
Female	3 (60%)	7 (41.2%)	47 (60.3%)	57 (57%)	
Ethnicity					
Black	2 (40%)	13 (76.5%)	54 (69.2%)	69 (69%)	0.29
Hispanic	2 (40%)	4 (23.5%)	21 (26.9%)	27 (27%)	
Asian	1 (20%)	0 (0%)	1 (1.3%)	2 (2%)	
Missing	0 (0%)	0 (0%)	2 (2.6%)	2 (2%)	
ATLL type					
Acute	1 (20%)	9 (52.9%)	57 (73.1%)	67 (67%)	**0.001**
Lymphomatous	4 (80%)	8 (47.1%)	10 (12.8%)	22 (22%)	
Missing	0 (0%)	0 (0%)	11 (14.1%)	11 (11%)	

Transplant only	Auto *n* = 5	Allo *n* = 17	Total *n* = 22	*p* value
Year of transplant	2015 (2005–2019) [2007–2016]	2016 (2012–2020) [2013–2018]	2015.5 (2005–2020) [2013–2018]	0.07
Lines of therapy before transplant	1 (1–3) [1, 2]	2 (1–4) [1–3]	2 (1–4) [1–3]	0.28
Diagnosis to transplant (months)	6 (4–7) [6, 7]	9 (4–378) [6–15]	7.5 (4–378) [6–11.75]	0.51
Follow-up (months)			65 (14–101) [36.75–96]	
Status at transplant				
CR1	2 (40%)	8 (47.1%)	10 (45.5%)	0.81
PR	1 (20%)	3 (17.6%)	4 (18.2%)	
PD/relapsed	2 (40%)	6 (35.3%)	8 (36.3%)	
Karnofsky score				
< 90	2 (40%)	3 (17.6%)	5 (22.7%)	0.18
≥ 90	2 (40%)	13 (76.5%)	15 (68.2%)	
Missing	1 (20%)	1 (5.9%)	2 (9.1%)	
Therapy group				
Chemotherapy only	4 (80%)	10 (58.8%)	14 (63.6%)	0.61
Chemotherapy + AZT/IFN	1 (20%)	7 (41.2%)	8 (36.4%)	

*CR* complete remission, *PR* partial remission, *PD* progressive disease

Twenty-two patients (45% female; median age 55.5 years [range 39–74]; 68% Blacks and 29% Hispanic) underwent transplant (Tables 1 and 2) with a median follow-up of alive patients of 65 months (IQR 24–95). Almost half (45%) of the patients were transplanted in CR1, 18% had achieved partial remission (PR), and 36% had progressive or relapsed disease. Transplanted patients had a median of 2 prior treatment lines (range 1–4) and were transplanted at a median of 7.5 months after diagnosis. Forty-five percent had acute ATLL, and the rest had a lymphomatous subtype. The KPS was ≥ 90 in 68% of patients, and 77% were CMV positive.

**Table 2:** Transplant characteristics

Transplant Characteristics	*n* = 22
	*N* (%)
Transplant type	
Allo	17 (77%)
Auto	5 (23%)
Donor	*n* = 17
Matched sibling	7 (41%)
10/10 Matched unrelated	1 (6%)
9/10 Matched unrelated	2 (12%)
Haploidentical	6 (35%)
Syngeneic	1 (6%)
Source of stem cells	*n* = 17
Bone marrow	3 (18%)
Peripheral blood	11 (65%)
Bone marrow + Peripheral blood	1 (6%)
Missing	2 (12%)
Donor gender	*n* = 16 (excluding syngeneic)
Male	7 (44%)
Female	5 (31%)
Missing	4 (25%)
Female to male	*n* = 16 (excluding syngeneic)
No	13 (81%)
Yes	2 (13%)
Missing	1 (6%)
Donor CMV serology	*n* = 17
CMV +	4 (24%)
CMV –	1 (6%)
Missing	12 (71%)
Donor HTLV serology	*n* = 17
HTLV +	3 (18%)
HTLV −	2 (12%)
Missing	12 (71%)
In vivo T-cell depletion	*n* = 16 (excluding syngeneic)
No	14 (88%)
Yes	1 (6%)
Missing	1 (6%)
Engraftment	*n* = 22
No	2 (9%)
Yes	20 (91%)
Conditioning	n = 22
RIC	15 (88%)
MAC	2 (12%)
TBI	*n* = 22
No	16 (73%)
Yes	6 (27%)
Conditioning details	*n* = 22
Flu-Mel	6 (27%)
Flu-Mel-ATG	2 (9%)
Flu-Cy-TBI	5 (23%)
Flu-Bu	2 (9%)
Cy-TBI	1 (5%)
BEAM	6 (27%)
Post-transplant cyclophosphamide	*n* = 16 (excluding syngeneic)
No	11 (69%)
Yes	5 (31%)
GVHD prophylaxis	*n* = 16 (excluding syngeneic)
Tacrolimus-Methotrexate	10 (63%)
Tacrolimus-Mycophenolate	6 (38%)

*CMV* Cytomegalovirus, *HTLV* Human T-lymphotropic virus, *MAC* Myeloablative conditioning, *RIC* Reduced intensity conditioning, *TBI* Total Body Irradiation, *Flu* Fludarabine, *Mel* Melphalan, *ATG* anti-thymocyte globulin, *Cy* cyclophosphamide, *BEAM* BCNU, etoposide, Ara‐C, and melphalan, *GVHD* Graft-vs-host disease

Seventeen patients underwent allo-SCT and five underwent ASCT. Allo-SCT patients received MAC in 12% of cases, and 27% of all patients received TBI. Donors for allo-SCT were 41% MSD, 18% MUD, and 35% haplo, and 65% of patients received PB stem cells, with one additional patient (6%) receiving both PB and BM cells. Only two male patients received cells from a female donor, and only three donors were documented HTLV1 positive and four CMV positive. In vivo T cell depletion (TCD) was only used in one case. GVHD prophylaxis was primarily tacrolimus-methotrexate (63%) with the rest receiving tacrolimus-mycophenolate; 31% of patients received post-transplant cyclophosphamide (PTCY).

### Transplant Outcomes

The 1-year RI was 43% and NRM 28%, while PFS and OS were 41% and 50%, respectively (Table 3). Three-year RI and NRM were 51% and 37%, respectively, and PFS and OS were 31% and 35%, respectively. Day + 180 acute GVHD grades II-IV and III-IV were encountered in 43% and 21% of patients who received allo-SCT, respectively, whereas the 1-year cumulative incidence of chronic and extensive GVHD were 50% and 36%, respectively (Table 4). Allo-SCT patients had 1-year and 3-years GRFS of 27% and 20%, respectively. Ten patients died after allo-SCT from primary disease (50%), infections (20%), GVHD (10%) and veno-occlusive disease (10%), and four ASCT patients died of disease progression (100%) (Table 5).

**Table 3:** Univariate analysis

Outcome from diagnosis		1 year	3 years
		OS	OS
Auto + Allo + No Transplant (*n* = 100)			
Whole group		42% [33–54]	20% [12–32]
Age at transplant	< Median	45% [33–63]	28% [17–47]
	≥ Median	38% [26–57]	11% [4–30]
	*p* value	0.44	0.15
Patient’s gender	Female	41% [29–57]	16% [8–35]
	Male	44% [30–63]	24% [13–44]
	*p* value	0.79	0.63
Ethnicity	Asian	0% [NA–NA]	0% [NA–NA]
	Black	45% [34–60]	24% [15–40]
	Hispanic	39% [24–65]	12% [3–41]
	*p* value	0.07	0.07
ATLL subtype	Acute	32% [22–48]	11% [5–27]
	Lymphomatous	71% [54–94]	42% [25–73]
	*p* value	**0.004**	**0.004**
Transplant type (*n* = 67, early mortality excluded in non-transplant group)	Allo	76% [59–100]	47% [28–78]
	Auto	60% [29–100]	20% [3–100]
	No transplant	47% [34–65]	15% [6–41]
	*p* value	**0.01**	**0.01**
No Transplant (no exclusions)		32% [22–46]	11% [4–28]

Outcome from transplant		1 year				3 years			
		RI	NRM	PFS	OS	RI	NRM	PFS	OS
Auto + Allo (*n* = 22)									
Whole group		43% [15–62]	28% [2–46]	41% [25–68]	50% [33–76]	51% [19–70]	37% [6–57]	31% [16–58]	35% [20–63]
Age at transplant	< Median	42% [0–67]	19% [0–40]	45% [24–87]	55% [32–94]	42% [0–67]	35% [0–62]	36% [17–79]	36% [17–79]
	≥ Median	43% [0–67]	35% [0–62]	36% [17–79]	45% [24–87]	62% [0–86]	35% [0–62]	24% [8–74]	34% [14–81]
	*p* value	0.91	0.6	0.67	0.68	0.62	0.86	0.62	0.89
Patient’s gender	Female	33% [0–58]	10% [0–27]	60% [36–100]	60% [36–100]	47% [0–72]	25% [0–50]	40% [19–85]	40% [19–85]
	Male	52% [5–76]	46% [0–73]	25% [9–67]	42% [21–81]	52% [5–76]	46% [0–73]	25% [9–67]	33% [15–74]
	*p* value	0.36	0.2	0.12	0.39	0.5	0.32	0.24	0.5
Year of transplant	< Median	48% [0–73]	28% [0–51]	36% [17–79]	55% [32–94]	48% [0–73]	28% [0–51]	36% [17–79]	45% [24–87]
	≥ Median	39% [0–62]	26% [0–52]	45% [24–87]	45% [24–87]	59% [0–84]	44% [0–73]	23% [7–74]	23% [7–74]
	*p* value	0.82	0.58	0.59	0.83	0.86	0.92	0.95	0.56
Diagnosis to transplant	< Median	42% [12–62]	17% [0–33]	47% [29–76]	58% [39–85]	50% [17–70]	27% [0–48]	36% [19–66]	41% [24–71]
	≥ Median	50% [0–87]	100% [NA–NA]	0% [NA–NA]	0% [NA–NA]	50% [0–87]	100% [NA–NA]	0% [NA–NA]	0% [NA–NA]
	*p* value	0.97	0.08	0.27	0.07	0.97	0.08	0.27	0.07
Ethnicity	Asian	100% [NA–NA]	0% [0–0]	0% [NA–NA]	0% [NA–NA]	100% [NA–NA]	0% [0–0]	0% [NA–NA]	0% [NA–NA]
	Black	32% [0–54]	21% [0–39]	53% [33–86]	60% [40–91]	43% [4–66]	32% [0–54]	38% [20–74]	45% [25–80]
	Hispanic	60% [0–86]	58% [0–90]	17% [3–100]	33% [11–100]	60% [0–86]	58% [0–90]	17% [3–100]	17% [3–100]
	*p* value	**0.001**	0.37	**0.01**	0.1	**0.001**	0.46	**0.02**	0.06
Karnofsky	< 90	62% [0–92]	40% [0–71]	20% [3–100]	40% [14–100]	62% [0–92]	100% [NA–NA]	0% [NA–NA]	0% [NA–NA]
	≥ 90	29% [0–50]	24% [0–45]	53% [33–86]	60% [40–91]	39% [4–61]	24% [0–45]	46% [26–80]	53% [32–86]
	*p* value	0.22	0.15	0.06	0.23	0.22	**0.02**	**0.01**	**0.01**
ATLL subtype	Acute	49% [0–74]	21% [0–44]	40% [19–85]	40% [19–85]	66% [2–88]	41% [0–69]	20% [6–69]	20% [6–69]
	Lymphomatous	39% [0–62]	32% [0–57]	42% [21–81]	58% [36–94]	39% [0–62]	32% [0–57]	42% [21–81]	50% [28–88]
	*p* value	0.7	0.85	0.86	0.35	0.45	0.81	0.46	0.2
Number of prior lines of therapy	< Median	43% [0–70]	12% [0–33]	50% [25–100]	62% [37–100]	57% [0–82]	12% [0–33]	38% [15–92]	38% [15–92]
	≥ Median	41% [5–64]	37% [0–62]	36% [18–72]	43% [23–78]	41% [5–64]	53% [1–78]	27% [11–66]	34% [16–72]
	*p* value	0.74	0.37	0.41	0.39	0.98	0.22	0.42	0.68
Status at transplant	Active	55% [3–79]	39% [0–64]	25% [9–67]	33% [15–74]	78% [0–95]	39% [0–64]	12% [2–68]	22% [7–69]
	CR1	30% [0–53]	14% [0–37]	60% [36–100]	70% [47–100]	30% [0–53]	29% [0–55]	50% [27–93]	50% [27–93]
	*p* value	0.16	0.1	**0.03**	**0.04**	0.07	0.18	**0.02**	0.08
Transplant type	Allo	30% [0–50]	32% [4–53]	47% [28–78]	53% [34–83]	41% [3–64]	42% [8–64]	34% [17–67]	40% [22–72]
	Auto	80% [0–97]	0% [0–0]	20% [3–100]	40% [14–100]	80% [0–97]	0% [0–0]	20% [3–100]	20% [3–100]
	*p* value	**0.03**	0.28	0.29	0.67	0.053	0.26	0.41	0.43

Outcome from transplant		1 year				3 years			
		RI	NRM	PFS	OS	RI	NRM	PFS	OS
Allo only (*n* = 17)									
Female to male transplant	No	18% [0–38]	30% [0–51]	57% [36–90]	57% [36–90]	32% [0–57]	40% [4–63]	41% [21–78]	41% [21–78]
	Yes	100% [NA–NA]	0% [0–0]	0% [NA–NA]	50% [13–100]	100% [NA–NA]	0% [0–0]	0% [NA–NA]	50% [13–100]
	*p* value	**0.0001**	0.5	0.06	0.84	**0.0001**	0.5	0.06	0.92
Stem cells source	BM	33% [0–70]	0% [0–0]	67% [30–100]	67% [30–100]	67% [0–93]	0% [0–0]	33% [7–100]	33% [7–100]
	PB	37% [0–62]	42% [0–67]	36% [17–79]	45% [24–87]	37% [0–62]	42% [0–67]	36% [17–79]	45% [24–87]
	*p* value	0.88	0.25	0.36	0.52	0.65	0.25	0.7	0.95
Donor	Haploidentical	40% [0–71]	17% [0–42]	50% [22–100]	50% [22–100]	70% [0–94]	17% [0–42]	25% [5–100]	25% [5–100]
	Matched sibling	33% [0–62]	54% [0–83]	29% [9–92]	43% [18–100]	33% [0–62]	54% [0–83]	29% [9–92]	43% [18–100]
	Matched unrelated	0% [0–0]	33% [0–70]	67% [30–100]	67% [30–100]	0% [0–0]	67% [0–93]	33% [7–100]	33% [7–100]
	*p* value	0.33	0.35	0.32	0.61	0.26	0.24	0.67	0.84
Conditioning	RIC	32% [0–54]	30% [0–52]	47% [27–80]	53% [33–86]	43% [4–66]	40% [4–63]	33% [16–68]	40% [22–74]
	MAC	0% [0–0]	50% [0–87]	50% [13–100]	50% [13–100]	0% [0–0]	50% [0–87]	50% [13–100]	50% [13–100]
	*p* value	0.55	0.45	0.84	0.73	0.55	0.45	0.84	0.73
TBI	No	41% [9–62]	25% [0–46]	44% [25–76]	56% [37–87]	41% [9–62]	37% [0–62]	36% [19–71]	43% [24–76]
	Yes	50% [0–81]	33% [0–62]	33% [11–100]	33% [11–100]	75% [0–95]	33% [0–62]	17% [3–100]	17% [3–100]
	*p* value	0.93	0.46	0.69	0.34	0.64	0.66	0.52	0.28
PTCY	No	22% [0–45]	40% [0–65]	45% [24–87]	55% [32–94]	22% [0–45]	52% [6–76]	36% [17–79]	45% [24–87]
	Yes	50% [0–81]	20% [0–48]	40% [14–100]	40% [14–100]	100% [NA–NA]	20% [0–48]	0% [NA–NA]	0% [NA–NA]
	*p* value	0.55	0.57	0.99	0.77	0.18	0.49	0.68	0.45

In univariate analysis comparing outcomes of Allo vs auto vs non-transplanted patients, 37 patients with early mortality < 4 months from diagnosis in the non-transplant group were excluded to account for immortal-time bias and hence *n* = 63 for that comparison only

*RI* relapse incidence, *NRM* non-relapse mortality, *PFS* progression-free survival, *OS* overall survival, *CR* complete remission, *BM* bone marrow, *PB* peripheral blood, *MAC* myeloablative conditioning, *RIC* reduced intensity conditioning, *TBI* Total Body Irradiation, *PTCY* Post-transplant cyclophosphamide

Bold *p*-values are significant *p*-values less than 0.05

**Table 4:** Univariate analysis

	180 days		1 year			3 years
	aGVHD II-IV	aGVHD III-IV	cGVHD	Extensive cGVHD	GRFS	GRFS
Allo only	43% [10–64]	21% [0–40]	50% [16–70]	36% [5–56]	27% [12–62]	20% [7–55]
Age at transplant						
< Median	57% [0–82]	43% [0–70]	86% [12–98]	57% [0–82]	29% [9–92]	29% [9–92]
≥ Median	29% [0–55]	0% [0–0]	14% [0–37]	14% [0–37]	25% [8–83]	12% [2–78]
*p* value	0.3	0.06	**0.01**	0.11	0.85	0.85
Patient’s gender						
Female	40% [0–71]	20% [0–48]	40% [0–71]	40% [0–71]	33% [11–100]	17% [3–100]
Male	44% [0–69]	22% [0–45]	56% [8–79]	33% [0–58]	22% [7–75]	22% [7–75]
*p* value	0.88	0.93	0.59	0.81	0.94	0.94
Year of transplant						
< Median	50% [0–78]	33% [0–62]	67% [0–89]	50% [0–78]	14% [2–88]	14% [2–88]
≥ Median	38% [0–63]	12% [0–33]	38% [0–63]	25% [0–50]	38% [15–92]	25% [8–83]
*p* value	0.65	0.36	0.3	0.35	0.22	0.22
Diagnosis to transplant						
< Median	67% [0–89]	33% [0–62]	50% [0–78]	33% [0–62]	43% [18–100]	29% [9–92]
≥ Median	25% [0–50]	12% [0–33]	50% [0–75]	38% [0–63]	12% [2–78]	12% [2–78]
* p* value	0.13	0.36	1	0.88	0.49	0.49
Ethnicity						
Black	33% [1–55]	25% [0–46]	50% [12–72]	42% [6–64]	25% [9–67]	17% [5–59]
Hispanic	100% [NA–NA]	0% [0–0]	50% [0–87]	0% [0–0]	33% [7–100]	33% [7–100]
*p* value	0.09	0.44	1	0.27	0.74	0.74
Karnofsky						
< 90	100% [NA–NA]	100% [NA–NA]	100% [NA–NA]	100% [NA–NA]	0% [NA–NA]	0% [NA–NA]
≥ 90	27% [0–49]	0% [0–0]	36% [1–59]	18% [0–38]	36% [17–79]	27% [10–72]
*p* value	0.06	**0.001**	0.11	**0.03**	**0.004**	**0.004**
ATLL subtype						
Acute	67% [16–87]	33% [0–58]	56% [8–79]	44% [0–69]	22% [7–75]	11% [2–71]
Lymphomatous	0% [0–0]	0% [0–0]	40% [0–71]	20% [0–48]	33% [11–100]	33% [11–100]
*p* value	**0.02**	0.16	0.59	0.38	0.64	0.64
Number of prior lines of therapy						
< Median	60% [0–86]	20% [0–48]	40% [0–71]	20% [0–48]	60% [29–100]	40% [14–100]
≥ Median	33% [0–58]	22% [0–45]	56% [8–79]	44% [0–69]	10% [2–64]	10% [2–64]
*p* value	0.35	0.93	0.59	0.38	0.09	0.09
Status at transplant						
Active	43% [0–70]	29% [0–55]	43% [0–70]	43% [0–70]	12% [2–78]	0% [NA–NA]
CR1	43% [0–70]	14% [0–37]	57% [0–82]	29% [0–55]	43% [18–100]	43% [18–100]
*p* value	1	0.53	0.61	0.59	**0.03**	**0.03**
Female to male transplant						
No	45% [6–68]	18% [0–38]	55% [13–76]	36% [1–59]	33% [15–74]	25% [9–67]
Yes	50% [0–87]	50% [0–87]	50% [0–87]	50% [0–87]	0% [NA–NA]	0% [NA–NA]
*p* value	0.91	0.35	0.91	0.73	0.33	0.33
Stem Cells Source						
BM	67% [0–93]	0% [0–0]	0% [0–0]	0% [0–0]	67% [30–100]	33% [7–100]
PB	25% [0–50]	25% [0–50]	62% [8–85]	50% [0–75]	11% [2–71]	11% [2–71]
*p* value	0.22	0.36	0.08	0.14	0.15	0.15
Donor						
Haploidentical	50% [0–78]	0% [0–0]	33% [0–62]	17% [0–42]	33% [11–100]	17% [3–100]
Matched sibling	33% [0–62]	33% [0–62]	50% [0–78]	33% [0–62]	33% [11–100]	33% [11–100]
Matched unrelated	50% [0–87]	50% [0–87]	100% [NA–NA]	100% [NA–NA]	0% [NA–NA]	0% [NA–NA]
*p* value	0.55	0.09	0.12	**0.04**	**0.01**	**0.01**
Conditioning						
RIC	42% [6–64]	17% [0–35]	42% [6–64]	25% [0–46]	31% [14–70]	23% [9–62]
MAC	50% [0–87]	50% [0–87]	100% [NA–NA]	100% [NA–NA]	0% [NA–NA]	0% [NA–NA]
*p* value	0.83	0.31	0.14	**0.048**	0.12	0.12
TBI						
No	25% [0–50]	25% [0–50]	62% [8–85]	50% [0–75]	22% [7–75]	22% [7–75]
Yes	67% [0–89]	17% [0–42]	33% [0–62]	17% [0–42]	33% [11–100]	17% [3–100]
* p* value	0.13	0.72	0.3	0.21	0.53	0.53
PTCY						
No	44% [0–69]	33% [0–58]	67% [16–87]	44% [0–69]	30% [12–77]	30% [12–77]
Yes	40% [0–71]	0% [0–0]	20% [0–48]	20% [0–48]	20% [3–100]	0% [NA–NA]
*p* value	0.88	0.16	0.11	0.38	0.79	0.79

*aGVHD* acute graft vs host disease, *cGVHD* chronic graft vs host disease, *GRFS* GVHD and relapse free survival, *CR* complete remission, *BM* bone marrow, *PB* peripheral blood, *MAC* myeloablative conditioning, *RIC* reduced intensity conditioning, *TBI* Total Body Irradiation, *PTCY* post-transplant cyclophosphamide

Bold *p*-values are significant *p*-values less than 0.05

**Table 5:** Cause of death

Cause of death	Allo-SCT	ASCT
	*n* = 10	*n* = 4
	*N* (%)	*N* (%)
Original disease	5 (50%)	4 (100%)
Infection	2 (20%)	0 (0%)
GVHD	1 (10%)	0 (0%)
Veno-occlusive disease	1 (10%)	0 (0%)
Missing	1 (10%)	0 (0%)

In the univariate analysis (Tables 3 and 4) gender, year of transplant, time from diagnosis to transplant, number of prior lines of therapy, stem cell source, TBI and PTCY use did not affect any of the transplant outcomes (RI, NRM, PFS, OS, acute and chronic GVHD, GRFS). On the other hand, at least one outcome was affected by each of the following variables: transplant type, disease subtype, patient age and ethnicity, KPS, status at transplant, female to male donor, conditioning intensity, and donor type.

**Fig. 1:**
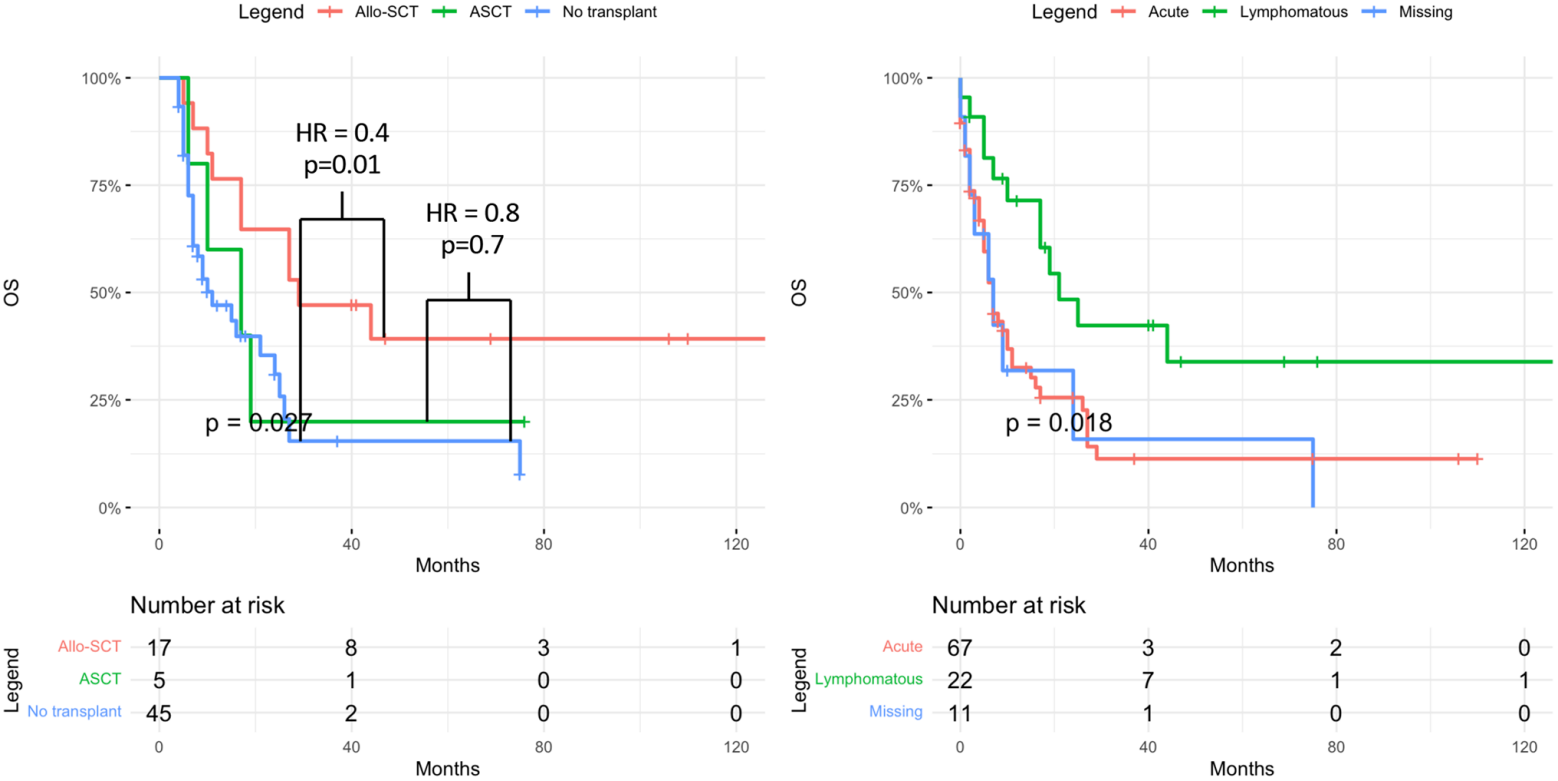
Overall survival post-diagnosis in ATLL patients undergoing allo-SCT vs ASCT vs no transplant and by clinical subtype. Patients with early mortality <4 months in the non-transplant group (*n*=33) were excluded from first graph to account for immortal-time bias

In univariate, landmark analysis excluding non-transplanted patients with early mortality within 4 months who were unable to make it to transplant, there was a significant difference in outcome with 1-year OS of 76% in allo, 60% in auto and 47% in the non-transplant group (*p* = 0.01), and 3-year OS of 47% in allo, 30% in auto, and 15% in the non-transplant group (*p* = 0.01) (Table 3, Fig. 1). Furthermore, univariate OS HR of allo-SCT versus no transplant was 0.4 with *p* = 0.01 while ASCT versus no transplant yielded a HR of 0.8 with *p* = 0.7. The use of ASCT was associated with significantly increased short-term relapse incidence compared to allo-SCT, with 1-year RI of 80% versus 30% (*p* = 0.03). Allo-SCT was associated with higher, though not statistically significant, NRM, which could explain the lack of any difference in short- or long-term OS (1-year OS 53% in allo versus 40% in auto, *p* = 0.67; 3-year OS 40% in allo versus 20% in auto, *p* = 0.43).

**Fig. 2:**
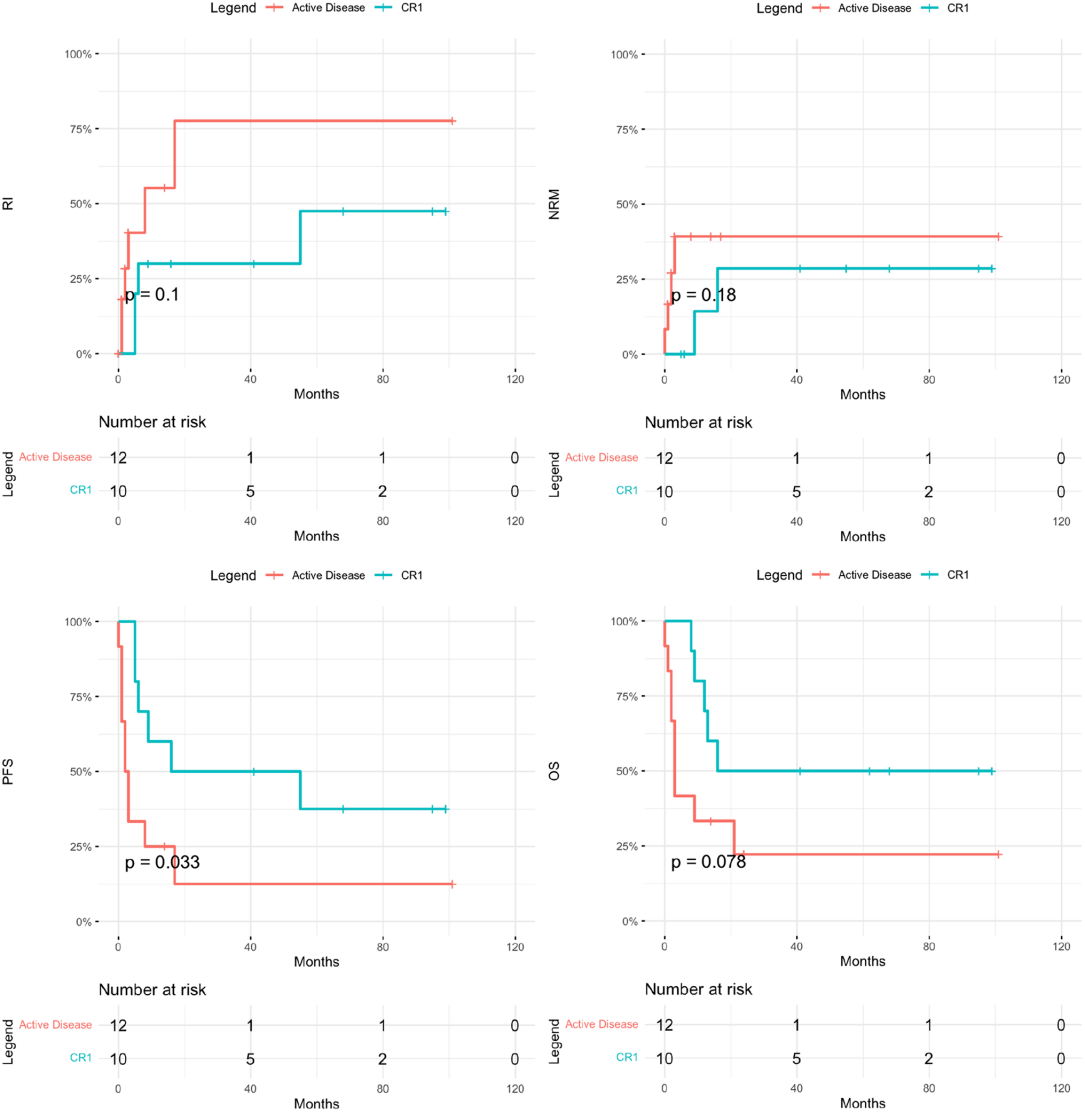
Post-transplant outcomes of ATLL patients in first complete remission (CR1) vs advanced disease

**Fig. 3:**
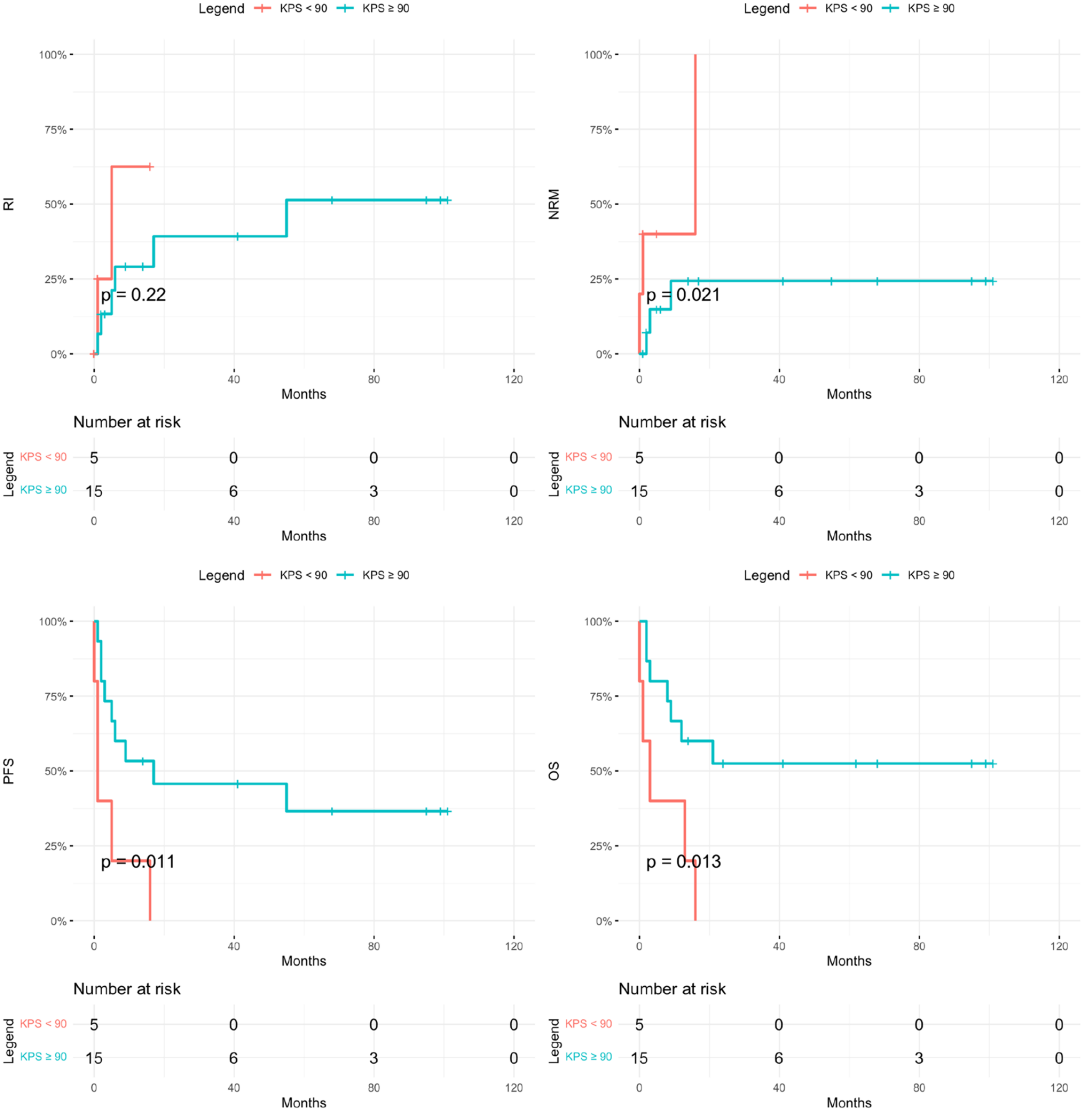
Post-transplant outcomes of ATLL patients with good performance status vs bad baseline status

While acute ATLL was associated with worse outcome in the entire cohort, irrespective of transplant status (Fig. 1), with 1-year OS of 32% vs. 71%, *p* = 0.004, and 3-year OS of 11% versus 42%, *p* = 0.004, within the transplant group, disease subtype did not impact any primary outcomes including RI, NRM, PFS and OS, except for rates of low grade aGVHD (67% in acute vs. 0% in lymphomatous, *p* = 0.02). As expected, patients in CR1 had significantly better 3-year outcomes compared to advanced disease with relapse rates of 30% vs. 78% (*p* = 0.07), PFS of 50% vs. 12% (*p* = 0.02), OS of 50% vs. 22% (*p* = 0.08), and GRFS of 43% vs. 0% (*p* = 0.03) (Fig. 2). Patients with KPS ≥ 90 had a significantly lower NRM (24%) compared to 100% in those with a KPS < 90 (*p* = 0.02), which also translated into improved PFS (46% vs. 0%, *p* = 0.01) and OS (53% vs. 0%, *p* = 0.01) (Fig. 3). They also had lower rates of acute and chronic GVHD, resulting in significantly improved GRFS (27% vs. 0%, *p* = 0.004). While choice of donor did not affect survival, it had an impact on GVHD incidence, translating into 3-year GRFS of 33% in MSD vs. 17% in haplo and 0% in MUD (*p* = 0.01).

Despite the small sample size, we performed an exploratory multivariate analysis (MVA) (Table 6). When including the entire patient cohort, after adjusting for disease subtype, allo-SCT resulted in a significantly improved OS, compared to no transplant, with an HR of 0.4 (*p* = 0.05). Similar to the finding in univariate analysis, ASCT had no visible impact on outcome, with an HR of 1 (*p* = 0.7). Among the transplanted patients only, KPS above 90 was strongly associated with improved NRM, PFS, OS and GRFS, with HRs of 0.05 (*p* = 0.02), 0.2 (*p* = 0.01), 0.2 (*p* = 0.03), and 0.1 (*p* = 0.01), respectively. Similarly, being in CR1 was also associated with improved outcomes affecting NRM, PFS and OS, with HRs of 0.05 (*p* = 0.03), 0.1 (*p* = 0.009) and 0.1 (0.02), respectively. Interestingly, patients of Hispanic race had more dismal outcomes compared to Black patients, with PFS HR of 5 (*p* = 0.04), and OS HR of 5 (*p* = 0.05).

**Table 6:** Exploratory multivariate analysis

Outcome	Variables	HR (95% CI)	*p* value
Auto + Allo + No Transplant			
OS	Lymphomatous vs Acute	0.5 (0.2–1)	0.09
	Allo vs No Transplant	0.4 (0.2–1)	**0.05**
	Auto vs No Transplant	1 (0.4–5)	0.7
Auto + Allo			
RI	Karnosky > 90	0.5 (0.05–5)	0.5
	CR1 vs other	0.2 (0.03–2)	0.2
	Hispanic vs Black	5 (0.7–40)	0.1
NRM	Karnosky > 90	0.05 (0.004–0.6)	**0.02**
	CR1 vs other	0.05 (0.003–0.8)	**0.03**
	Hispanic vs Black	4 (0.4–40)	0.2
PFS	Karnosky > 90	0.2 (0.04–0.7)	**0.01**
	CR1 vs other	0.1 (0.03–0.6)	**0.009**
	Hispanic vs Black	5 (1–20)	**0.04**
OS	Karnosky > 90	0.2 (0.03–0.9)	**0.03**
	CR1 vs other	0.1 (0.02–0.7)	**0.02**
	Hispanic vs Black	5 (1–20)	**0.05**
Allo only			
GRFS	Karnosky > 90	0.1 (0.02–0.7)	**0.01**
	CR1 vs other	0.4 (0.1–1)	0.1
	Hispanic vs Black	1 (0.3–7)	0.6

Patients with early mortality < 4 months in the non-transplant group were excluded from first graph to account for immortal-time bias

*HR* hazard ratio, *CI* confidence interval, *CR1* first complete remission, *RI* relapse incidence, *NRM* non-relapse mortality, *PFS* progression-free survival, *OS* overall survival, *GRFS* GVHD and relapse free survival

Bold *p*-values are significant *p*-values less than 0.05

## Discussion

Our data are in line with the reported literature on ATLL, particularly in its acute subtype, being associated with dismal long-term outcome [28]. ASCT is associated with increased short-term relapse compared to allo-SCT, making it a poor treatment choice [12–14]. Indeed, 80% of our patients undergoing ASCT relapsed within 1 year of transplant and died of disease progression. Importantly, no difference in outcome was noted when comparing ASCT to non-transplanted patients, with an HR of 0.8 (*p* = 0.7), further demonstrating why it should no longer be offered to ATLL patients.

This study highlights the importance of allo-SCT in this aggressive disease, and suggests that it should be offered early in the disease course, while patients still have good performance status, rather than postponing it as a salvage therapy. Good performance status and having achieved CR prior to transplant are known positive outcome predictors, and our findings here confirmed these concepts, with a complementary effect in patients with both good performance status and transplanted in CR who had 5-year PFS and OS of 47% and 62%, respectively. Allo-SCT can be offered to both acute and lymphomatous ATLL, as it appears to partially overcome the dismal outcome associated with acute disease, which usually responds poorly to conventional chemotherapy. In fact, no differences were noted between acute and lymphomatous subtypes across all outcome parameters, despite acute subtype having significantly worse outcome in the non-transplant group. Our patients had good long-term outcomes post allo-SCT even after 5 years of follow-up and over, with 40% OS from transplant. Importantly, we show that allo-SCT significantly improves survival compared to no transplant even in a landmark analysis which accounts for immortal-time bias, with allo-SCT recipients HR of 0.4 (*p* = 0.01) compared to non-transplant patients. This is again consistent with the literature and shows that allo-SCT is effective even in NA-ATLL which has worse overall outcome.

One of the major advantages of allo-SCT is thought to be attributed to its GVL effect. While we cannot directly measure the GVL effect, we know that developing GVHD post-transplant can be a good indirect surrogate. As such, when comparing the RI of patients who developed chronic GVHD post-allo to those who did not, at the 3-year mark, there was a notable difference in outcome, with only 17% RI in patients with chronic GVHD compared to 67% in those without it.

Unexpectedly, Hispanic patients in the transplant cohort had a worse outcome compared to Black patients, regardless of performance status and disease response, as evident in the MVA; yet, Hispanic ethnicity had no impact on survival when the analysis included non-transplant patients. This ethnic difference between Hispanic and Black patients which specifically impacts transplanted ATLL patients has not been previously reported and was contrary to findings in other settings [29]. The underlying cause is currently unknown, and could possibly be attributed to epidemiologic genetic variations in HTLV-1 subtypes between the two groups, as well as differences in anti-HTLV-1 immunity and/or variations in ATLL pathobiology between the two ethnic groups. Lack of compatible donors and barriers to early transplant in Hispanics may also be of critical importance, as previously published as well [30, 31]. Further investigation is required including immune and genomic profiling of these patients.

## Conclusion

The treatment of ATLL and particularly NA-ATLL remains challenging, with most patients relapsing and succumbing to the disease. Few options are available as salvage post-relapse, making SCT an attractive and potentially curative approach. ASCT has been studied in limited case series and was found to consistently lead to high relapse rates and overall poor outcome, while allo-SCT, when feasible, can induce long-term remission. Its use remains limited owing to its toxicity and patient’s usual poor performance status, as well as the disproportionate prevalence of HTLV1 in ethnic minorities, making it often challenging to find suitable donors. Our data are consistent with the literature, as almost all our patients were of either Hispanic or Black ethnicities, and carefully selected to have good performance status and having achieved CR at transplant. ASCT was associated with high relapse rates and, despite occasionally inducing long term remission, as observed in one of our patients who sustained remission after 68 months, consistent with previous reports [32], we do not recommend its routine use. Early allo-SCT is an effective treatment option, partially overcoming the dismal outcome of the acute ATLL subtype, and inducing long term remission in 40% of patients. Hispanic patients had worse outcome compared to Black patients when undergoing transplant. Although this could be attributed to lack of suitable donors or barriers to early transplant in this group, further studies are necessary to explain this difference.

## Data Availability

Data will be made available upon reasonable request.

## Abbreviations

ATLL: Adult T-cell leukemia/lymphoma
allo-SCT: Allogeneic stem-cell transplantation
ASCT: Autologous SCT
RI: Relapse incidence
NRM: Non-relapse mortality
PFS: Progression-free survival
OS: Overall survival
HTLV-1: Human T-cell lymphotropic virus type I
NA-ATLL: ATLL from north America
SCT: Stem-cell transplantation
CR: Complete response
MAC: Myeloablative conditioning
RIC: Reduced intensity conditioning
GVL: Graft-vs-leukemia
GVHD: Graft-vs-host-disease
DLI: Donor lymphocyte infusion
MSD: Matched related
MUD: Unrelated
haplo: Haploidentical
CB: Cord blood
BM: Bone marrow
PB: Peripheral blood
CMV: Cytomegalovirus
KPS: Karnofsky performance score
Bu: Busulfan
HR: Hazard ratio
CI: Confidence interval
PR: Partial remission
TCD: T cell depletion
PTCY: Post-transplant cyclophosphamide
MVA: Multivariate analysis

## References

[1] Yamamoto N, Hinuma Y (1982). Antigens in an adult T-cell leukemia virus-producer cell line: reactivity with human serum antibodies. Int J Cancer.

[2] Poiesz BJ, Ruscetti FW, Gazdar AF, Bunn PA, Minna JD, Gallo RC (1980). Detection and isolation of type C retrovirus particles from fresh and cultured lymphocytes of a patient with cutaneous T-cell lymphoma. Proc Natl Acad Sci USA.

[3] Tsukasaki K, Hermine O, Bazarbachi A, Ratner L, Ramos JC, Harrington W (2009). Definition, prognostic factors, treatment, and response criteria of adult T-cell leukemia-lymphoma: a proposal from an international consensus meeting. J Clin Oncol.

[4] Gessain A, Cassar O (2012). Epidemiological Aspects and World Distribution of HTLV-1 Infection. Front Microbiol.

[5] Shimoyama M (1991). Diagnostic criteria and classification of clinical subtypes of adult T-cell leukaemia-lymphoma. A report from the Lymphoma Study Group (1984–87). Br J Haematol.

[6] Takasaki Y, Iwanaga M, Imaizumi Y, Tawara M, Joh T, Kohno T (2010). Long-term study of indolent adult T-cell leukemia-lymphoma. Blood.

[7] Tsukasaki K, Utsunomiya A, Fukuda H, Shibata T, Fukushima T, Takatsuka Y (2007). VCAP-AMP-VECP compared with biweekly CHOP for adult T-cell leukemia-lymphoma: Japan Clinical Oncology Group Study JCOG9801. J Clin Oncol.

[8] Bazarbachi A, Plumelle Y, Carlos Ramos J, Tortevoye P, Otrock Z, Taylor G (2010). Meta-analysis on the use of zidovudine and interferon-alfa in adult T-cell leukemia/lymphoma showing improved survival in the leukemic subtypes. J Clin Oncol.

[9] Hodson A, Crichton S, Montoto S, Mir N, Matutes E, Cwynarski K (2011). Use of zidovudine and interferon alfa with chemotherapy improves survival in both acute and lymphoma subtypes of adult T-cell leukemia/lymphoma. J Clin Oncol.

[10] Zell M, Assal A, Derman O, Kornblum N, Battini R, Wang Y (2016). Adult T-cell leukemia/lymphoma in the Caribbean cohort is a distinct clinical entity with dismal response to conventional chemotherapy. Oncotarget.

[11] Shah UA, Chung EY, Giricz O, Pradhan K, Kataoka K, Gordon-Mitchell S (2018). North American ATLL has a distinct mutational and transcriptional profile and responds to epigenetic therapies. Blood.

[12] Tsukasaki K, Maeda T, Arimura K, Taguchi J, Fukushima T, Miyazaki Y (1999). Poor outcome of autologous stem cell transplantation for adult T cell leukemia/lymphoma: a case report and review of the literature. Bone Marrow Transplant.

[13] Phillips AA, Willim RD, Savage DG, Horwitz SM, Isola L, Zain JM (2009). A multi-institutional experience of autologous stem cell transplantation in North American patients with human T-cell lymphotropic virus type-1 adult T-cell leukemia/lymphoma suggests ineffective salvage of relapsed patients. Leuk Lymphoma.

[14] Bazarbachi A, Cwynarski K, Boumendil A, Finel H, Fields P, Raj K (2014). Outcome of patients with HTLV-1-associated adult T-cell leukemia/lymphoma after SCT: a retrospective study by the EBMT LWP. Bone Marrow Transplant.

[15] Marçais A, Suarez F, Sibon D, Bazarbachi A, Hermine O (2012). Clinical trials of adult T-cell leukaemia/lymphoma treatment. Leuk Res Treatment.

[16] Utsunomiya A, Miyazaki Y, Takatsuka Y, Hanada S, Uozumi K, Yashiki S (2001). Improved outcome of adult T cell leukemia/lymphoma with allogeneic hematopoietic stem cell transplantation. Bone Marrow Transplant.

[17] Kami M, Hamaki T, Miyakoshi S, Murashige N, Kanda Y, Tanosaki R (2003). Allogeneic haematopoietic stem cell transplantation for the treatment of adult T-cell leukaemia/lymphoma. Br J Haematol.

[18] Fukushima T, Miyazaki Y, Honda S, Kawano F, Moriuchi Y, Masuda M (2005). Allogeneic hematopoietic stem cell transplantation provides sustained long-term survival for patients with adult T-cell leukemia/lymphoma. Leukemia.

[19] Okamura J, Utsunomiya A, Tanosaki R, Uike N, Sonoda S, Kannagi M (2005). Allogeneic stem-cell transplantation with reduced conditioning intensity as a novel immunotherapy and antiviral therapy for adult T-cell leukemia/lymphoma. Blood.

[20] Hishizawa M, Kanda J, Utsunomiya A, Taniguchi S, Eto T, Moriuchi Y (2010). Transplantation of allogeneic hematopoietic stem cells for adult T-cell leukemia: a nationwide retrospective study. Blood.

[21] Uike N, Tanosaki R, Utsunomiya A, Choi I, Okamura J (2011). Can allo-SCT with RIC cure ATLL? Long-term survivors with excellent PS and with heterogenous HTLV-1 proviral load level. Retrovirology.

[22] Itonaga H, Tsushima H, Taguchi J, Fukushima T, Taniguchi H, Sato S (2013). Treatment of relapsed adult T-cell leukemia/lymphoma after allogeneic hematopoietic stem cell transplantation: the Nagasaki Transplant Group experience. Blood.

[23] Ishida T, Hishizawa M, Kato K, Tanosaki R, Fukuda T, Takatsuka Y (2013). Impact of graft-versus-host disease on allogeneic hematopoietic cell transplantation for adult T cell leukemia-lymphoma focusing on preconditioning regimens: nationwide retrospective study. Biol Blood Marrow Transplant.

[24] Bacigalupo A, Ballen K, Rizzo D, Giralt S, Lazarus H, Ho V (2009). Defining the intensity of conditioning regimens: working definitions. Biol Blood Marrow Transplant.

[25] Glucksberg H, Storb R, Fefer A, Buckner CD, Neiman PE, Clift RA (1974). Clinical manifestations of graft-versus-host disease in human recipients of marrow from HL-A-matched sibling donors. Transplantation.

[26] Terwey TH, Vega-Ruiz A, Hemmati PG, Martus P, Dietz E, le Coutre P (2012). NIH-defined graft-versus-host disease after reduced intensity or myeloablative conditioning in patients with acute myeloid leukemia. Leukemia.

[27] Ruggeri A, Labopin M, Ciceri F, Mohty M, Nagler A (2016). Definition of GvHD-free, relapse-free survival for registry-based studies: an ALWP-EBMT analysis on patients with AML in remission. Bone Marrow Transplant.

[28] Malpica L, Pimentel A, Reis IM, Gotuzzo E, Lekakis L, Komanduri K (2018). Epidemiology, clinical features, and outcome of HTLV-1-related ATLL in an area of prevalence in the United States. Blood Adv.

[29] Ruiz JM, Steffen P, Smith TB (2013). Hispanic mortality paradox: a systematic review and meta-analysis of the longitudinal literature. Am J Public Health.

[30] Landry I (2021). Racial disparities in hematopoietic stem cell transplant: a systematic review of the literature. Stem Cell Investig.

[31] Adrianzen Herrera D, Kornblum N, Acuna-Villaorduna A, Sica RA, Shah U, Butler M (2019). Barriers to allogeneic hematopoietic stem cell transplantation for human T cell lymphotropic virus 1-associated adult T cell lymphoma-leukemia in the United States: experience from a large cohort in a major Tertiary Center. Biol Blood Marrow Transplant.

[32] Utsunomiya A, Tokunaga M, Nakano N, Fujiwara H, Miyamoto T, Ogata M (2022). Long-term follow-up of patients with ATL after autologous stem cell transplantation. Bone Marrow Transplant.

